# Energy Efficiency Optimization in Massive MIMO Secure Multicast Transmission

**DOI:** 10.3390/e22101145

**Published:** 2020-10-12

**Authors:** Bin Jiang, Linbo Qu, Yufei Huang, Yifei Zheng, Li You, Wenjin Wang

**Affiliations:** 1 National Mobile Communications Research Laboratory, Southeast University, Nanjing 210096, China; qlbdeyx@163.com (L.Q.); yufei_huang@seu.edu.cn (Y.H.); 220190685@seu.edu.cn (Y.Z.); liyou@seu.edu.cn (L.Y.); wangwj@seu.edu.cn (W.W.); 2 Key Laboratory of Dynamic Cognitive System of Electromagnetic Spectrum Space, Ministry of Industry and Information Technology, Ministry of Industry and Information Technology, Nanjing University of Aeronautics and Astronautics, Nanjing 210016, China; 3 Purple Mountain Laboratories, Nanjing 211100, China

**Keywords:** energy efficiency optimization, massive MIMO, privacy engineering, utility–privacy trade-off, statistical CSI, beam domain power allocation

## Abstract

Herein, we focus on energy efficiency optimization for massive multiple-input multiple-output (MIMO) downlink secure multicast transmission exploiting statistical channel state information (CSI). Privacy engineering in the field of communication is a hot issue under study. The common signal transmitted by the base station is multicast transmitted to multiple legitimate user terminals in our system, but an eavesdropper might eavesdrop this signal. To achieve the energy efficiency utility–privacy trade-off of multicast transmission, we set up the problem of maximizing the energy efficiency which is defined as the ratio of the secure transmit rate to the power consumption. To simplify the formulated nonconvex problem, we use a lower bound of the secure multicast rate as the molecule of the design objective. We then obtain the eigenvector of the optimal transmit covariance matrix into a closed-form, simplifying the matrix-valued multicast transmission strategy problem into a power allocation problem in the beam domain. By utilizing the Minorize-Maximize method, an iterative algorithm is proposed to decompose the secure energy efficiency optimization problem into a sequence of iterative fractional programming subproblems. By using Dinkelbach’s transform, each subproblem becomes an iterative problem with the concave objective function, and it can be solved by classical convex optimization. We guarantee the convergence of the two-level iterative algorithm that we propose. Besides, we reduce the computational complexity of the algorithm by substituting the design objective with its deterministic equivalent. The numerical results show that the approach we propose performs well compared with the conventional methods.

## 1. Introduction

With the great development of network information technology and the continuous improvement of wireless communication technology, modern society puts forward higher requirements for the current communication transmission. With the extensive amount of research, there are a number of breakthrough technologies, such as the multiple-input multiple-output (MIMO) technology [1]. This technology has great potential to improve both the spectrum efficiency and the energy efficiency of the system, which is the development direction of future 5G/B5G wireless communication systems [2,3]. In the long-term evolution (LTE) standard, the wireless multicast transmission is also widely studied. In wireless multicast transmission, one base station (BS) sends public information to multiple user terminals (UTs). That is to say, that the BS can effectively transmit group-oriented signals by utilizing an evolved multimedia broadcast multicast service (eMBMS) [4,5,6,7,8]. As a mature technology, wireless multicast technology has certain advantages in improving the network traffic and reducing the communication cost. The combination of wireless multicast and massive MIMO is widely concerned with improving the quality of service (QoS), owing to its performance of effectively shaping the multicast transmission signals [9,10,11,12].

Due to the openness of the wireless propagation media, security issues are critical indicators in wireless transmissions of all fields. To ensure the security of transmission, many works traditionally use key-based enciphering. However, this method meets its challenge for secure wireless communications with additional control signaling and feedback channel management. Therefore, the security and privacy of physical layer security in wireless channels have a wide range of research [13,14,15], thanks to its great ability to work in a keyless scenarios. In previous works, the physical layer security of massive MIMO unicast transmission has been studied [16,17,18,19]. Meanwhile, in some existing works, the physical layer security of multicast transmission is also considered. For example, secure multicast precoding has been investigated [20,21,22]. Besides, some works have studied the security by introducing artificial noise into the multicast transmission, e.g., in [23,24].

Energy efficiency is another important indicator of performance in designing wireless transmission, which has been widely concerned by both academia and industry [25,26]. About 5% of the energy consumption of the whole world is produced by information and communication technologies, owing to its connected devices and infrastructure constantly being consumed [27]. In the future of 5G/B5G development, energy demand will soon become unmanageable. Therefore, a promising answer to this issue is to find considerable metrics which can limit energy consumptions but meet the needs of secure and reliable communication. However, most of the works on secure multicast transmission are limited to optimizing the secure rate, and almost no energy efficiency issues are considered. Some existing works have investigated the energy efficiency of unicast transmission instead of multicast transmission [28,29,30]. In some previous works, the energy efficiency multicast transmission was investigated [31,32], but the issues of security were not solved well.

In some works, it is assumed that the legitimate UTs’ instantaneous channel state information (CSI) is known [17,21,22,23,33]. However, due to CSI’s capture/feedback overhead in frequency division duplex MIMO systems, there are some difficulties in obtaining instantaneous CSI at the BS. It is the reason why most of the previous work has assumed this. On the other hand, statistical CSI has enormous advantages over instantaneous CSI for BS to obtain. Because of its slower change, BS can get statistical CSI accurately [34]. Therefore, statistical CSI is more suitable for wireless transmission design in high mobility scenarios. Besides, as a vast antenna array with high spatial resolution, massive MIMO channel typically exhibits new statistical characteristics [35,36].

We study the energy efficiency of massive MIMO secure multicast transmission with the assumption that only the statistical CSI of legal UTs and the eavesdropper can be obtained at the BS. We summarize the contributions of the current paper as follows.

- We first considered a secure multicast transmission scenario, in which the BS can only obtain the statistical CSI of the legitimate UTs and eavesdropper, and formulate our energy efficiency maximization problem. A tight lower bound rate of secure multicast is presented to simplify the optimization objective. Therefore, our problem is transformed into finding the optimal solution of this equivalent problem.
- Via making the transmit signaling directions into a closed-form, we simplify the energy efficiency optimization of matrix-valued strategy design to a beam domain power allocation problem, reducing the optimization variables significantly.
- By invoking the Minorize–Maximize (MM) method and Dinkelbach’s transform, we propose a two-level iterative algorithm to solve the equivalent problem. In the outer iteration, we decompose the original problem into several fractional programming subproblems, where the numerator is concave and the denominator is linear. In the inner iteration, we introduce related variables to remove the denominator and transform each subproblem into a common convex optimization problem to obtain the optimal solution of the equivalent problem. Our simulation results reveal that this solution is almost equal to the original problem.
- According to the large-dimensional random matrix theory, we use the deterministic equivalent of the secure transmit rate instead of massive sample averaging to reduce the computational complexity, which is proved to be almost the same as the original result.

The rest of this paper is arranged as follows. Section 2 presents the massive MIMO channel model. In Section 3, we divide the algorithm into three parts. The energy efficiency of secure multicast transmission is investigated. The optimal energy efficiency multicast transmit covariance matrix is decomposed into eigenvectors and eigenvalues as pretreatment. We propose an iterative algorithm for some fractional programming subproblems. Each subproblem is also solved by the inner iterative algorithm of sequential beam domain power allocation. The last part is to simplify the complexity of the problem. In Section 4, numerical simulation results are given. Finally, we summarize the full work in Section 5.

In our work, the use of symbols is also an important part. The main adopted symbols are as follows.

- $C^{M\times N}$ denotes M×N dimensional complex-valued vector space and $R^{M\times N}$ denotes M×N dimensional real-valued vector space.
- ${\left[A\right]}_{m,n}$ denotes the (m,n) th element of matrix A.
- det {·} denotes the matrix determinant operation, tr {·} denotes the matrix trace operation, and diag {·} denotes the diagonalization of the matrix, respectively.
- $E\left\{\cdot \right\}$ denotes the expectation operation and ${\left[x\right]}^{+}$ denotes max$\left\{x,0\right\}$.
- ${\left(\cdot \right)}^{T}$, ${\left(\cdot \right)}^{*}$, ${\left(\cdot \right)}^{H}$ and ${\left(\cdot \right)}^{-1}$ denote the matrix transpose, conjugate, conjugate-transpose and inverse, respectively.
- ⊙ denotes the Hadamard product.
- X⪰0 denotes that X is a positive semidefinite matrix.

## 2. Channel Model

We consider a massive MIMO secure multicast downlink transmission system, in which public messages sent by the BS are transmitted to the legitimate UTs. There is one BS with *M* antenna transmits and *K* UTs, each with $N_{r}$ antennas. The public messages are secret to the eavesdropper with $N_{e}$ antennas.

$x\in C^{M\times 1}$ denotes the signal that is sent from the BS in multicast transmission. $E\left\{x\right\}=0$ and $E\left\{xx^{H}\right\}=Q\in C^{M\times M}$, where Q denotes the transmit covariance matrix. We model the signals received by legitimate UT *k* and the eavesdropper as

(1) $$\begin{array}{cc} y_{k} & =H_{k}x+n_{k}\in C^{N_{r}\times 1}, \end{array}$$

(2) $$\begin{array}{cc} y_{\mathrm{ev}} & =H_{\mathrm{ev}}x+n_{\mathrm{ev}}\in C^{N_{e}\times 1}, \end{array}$$

respectively, where $H_{k}$ and $H_{\mathrm{ev}}$ are defined as the channel matrices of BS-UT *k* and BS-eavesdropper, respectively.

We consider our MIMO channel model to follow jointly spatially correlated Rayleigh fading, which means there are joint correlation characteristics between the transmitter and the receivers [37]. Thus, the channel matrices $H_{k}$ and $H_{\mathrm{ev}}$ in (1) and (2) can be expressed as

(3) $$\begin{array}{cc} H_{k} & =U_{k}G_{k}V_{k}^{H}\in C^{N_{r}\times M}, \end{array}$$

(4) $$\begin{array}{cc} H_{\mathrm{ev}} & =U_{\mathrm{ev}}G_{\mathrm{ev}}V_{\mathrm{ev}}^{H}\in C^{N_{e}\times M}, \end{array}$$

respectively, where $G_{k}\in C^{N_{r}\times M}$ and $G_{\mathrm{ev}}\in C^{N_{e}\times M}$ are random matrices with zero mean and independent elements, which are defined as the channel matrices of BS-UT *k* and BS-eavesdropper in the beam domain, respectively [34,38]. $U_{k}\in C^{N_{r}\times N_{r}}$, $V_{k}\in C^{M\times M}$, $U_{\mathrm{ev}}\in C^{N_{e}\times N_{e}}$, and $V_{\mathrm{ev}}\in C^{M\times M}$ are unitary matrices of transmitter and receivers, respectively. The statistics of the beam domain channels can be described as

(5) $$\begin{array}{cc} \Omega_{k} & =E\left\{G_{k}⊙G_{k}^{*}\right\}\in R^{N_{r}\times M}, \end{array}$$

(6) $$\begin{array}{cc} \Omega_{\mathrm{ev}} & =E\left\{G_{\mathrm{ev}}⊙G_{\mathrm{ev}}^{*}\right\}\in R^{N_{e}\times M}, \end{array}$$

respectively. It can be proved that as the number of antennas in the BS, *M*, increases to infinity, the eigenvectors of the correlation matrices in (5) and (6) tend to be equal, which can be denoted by a deterministic unitary matrix V [38]. V is only effected by the topology of transmit array at the BS, which means the channel matrices can be rewritten as

(7) $$\begin{array}{cc} H_{k} & \overset{M\to \infty }{=}U_{k}G_{k}V^{H}, \end{array}$$

(8) $$\begin{array}{cc} H_{\mathrm{ev}} & \overset{M\to \infty }{=}U_{\mathrm{ev}}G_{\mathrm{ev}}V^{H}, \end{array}$$

respectively. It has been widely used in previous works and shows considerable accuracy for a finite number of BS antennas [35,36]. Thus, we adopt the channel models in (7) and (8) in this paper.

## 3. Energy Efficiency Optimization of Secure Multicast Transmission

We study the energy efficiency multicast optimization of secure massive MIMO in this section. We assume that the BS can only capture the statistical CSI of all legitimate UTs and the eavesdropper [34]. Besides, legitimate UTs and the eavesdropper can obtain the instantaneous CSI. Before constructing the energy efficiency model, we first analyze the secure transmit rate. The received signal transmission rate needs to be higher for any user terminal than that received by the eavesdropper. Focusing on delay-tolerant scenarios [39], the ergodic secure multicast rate can be expressed as

(9) $$\begin{array}{c} R_{\mathrm{se}}≜{\left[R_{\mathrm{mc}}-R_{\mathrm{ev}}\right]}^{+}, \end{array}$$

where the ergodic multicast rate $R_{\mathrm{mc}}$ is expressed as

(10) $$\begin{array}{cc} R_{\mathrm{mc}} & =\min_{k}\;E\left\{\log\det\left\{I_{N_{r}}+H_{k}QH_{k}^{H}\right\}\right\}\\ \; & =\min_{k}\;E\left\{\log\det\left\{I_{N_{r}}+G_{k}V^{H}QVG_{k}^{H}\right\}\right\}, \end{array}$$

and the ergodic rate for eavesdropper $R_{\mathrm{ev}}$ is expressed as

(11) $$\begin{array}{cc} R_{\mathrm{ev}} & =E\left\{\log\det\left\{I_{N_{e}}+H_{\mathrm{ev}}QH_{\mathrm{ev}}^{H}\right\}\right\}\\ \; & =E\left\{\log\det\left\{I_{N_{e}}+G_{\mathrm{ev}}V^{H}QVG_{\mathrm{ev}}^{H}\right\}\right\}. \end{array}$$

In (10) and (11), we exploit the identity $\det\left\{I+AB\right\}=\det\left\{I+BA\right\}$.

Meanwhile, we adopt a model of power consumption as follows. For large-scale MIMO systems, most of the power is consumed by the BS, we then focus mainly on the power consumption of the BS. Besides, the static and dynamic power consumption of the radio frequency chains is considered. Specifically, our power consumption model is adopted as

(12) $$\begin{array}{cc} P(Q) & ≜\zeta \mathrm{tr}\left\{Q\right\}+MP_{c}+P_{s}, \end{array}$$

where $\mathrm{tr}\left\{Q\right\}$ denotes the transmit power of multicast transmission. Note that the coefficient ζ≥1 is the reciprocal of the transmit amplifier drain efficiency, $P_{s}$ and $P_{c}$ are the static power consumption and the hardware dissipated power of the BS, respectively.

We denote *W* by the bandwidth of secure transmission, the energy efficiency of massive MIMO secure multicast transmission is formulated by the ratio of secure transmit rate to power consumption as follows,

(13) $$\begin{array}{c} {\mathrm{EE}}_{\mathrm{se}}=\frac{WR_{\mathrm{se}}}{P}. \end{array}$$

Our aim is optimizing the transmit covariance matrix Q, leading to maximizing the ${\mathrm{EE}}_{\mathrm{se}}$ of downlink secure multicast in (13). The problem is addressed as

(14) $$\begin{array}{cc} \underset{Q}{\arg\max}\; & \frac{WR_{\mathrm{se}}}{P},\\ s.t.\; & \mathrm{tr}\left\{Q\right\}\leq P_{\max},Q⪰0, \end{array}$$

where $P_{\max}$ denotes the maximum multicast power budget at the transmitter.

$R_{\mathrm{ev}}$ in (11) can get its upper bound via using Jensen’s inequality, which is expressed as follows,

(15) $$\begin{array}{cc} R_{\mathrm{ev}} & \leq R_{\mathrm{ev},\mathrm{ub}}≜\log\det\left\{I_{N_{e}}+\underset{≜A_{\mathrm{ev}}\left(V^{H}QV\right)}{\underset{︸}{E\left\{G_{\mathrm{ev}}V^{H}QVG_{\mathrm{ev}}^{H}\right\}}}\right\}, \end{array}$$

where $A_{\mathrm{ev}}\left(X\right)≜E\left\{G_{\mathrm{ev}}XG_{\mathrm{ev}}^{H}\right\}\in C^{N_{e}\times N_{e}}$ denotes a function of matrix-valued form, which can output a diagonal matrix. Its *i*th diagonal element can be expressed as

(16) $$\begin{array}{c} {\left[A_{\mathrm{ev}}\left(X\right)\right]}_{i,i}=\mathrm{tr}\left\{\mathrm{diag}\left\{{\left({\left[\Omega_{\mathrm{ev}}\right]}_{i,:}\right)}^{T}\right\}X\right\}. \end{array}$$

By employing the function in (15), the secure multicast rate in (9) can obtain its lower bound as

(17) $$\begin{array}{c} R_{\mathrm{se}}\geq R_{\mathrm{se},\mathrm{lb}}≜{\left[R_{\mathrm{mc}}-R_{\mathrm{ev},\mathrm{ub}}\right]}^{+}. \end{array}$$

For a vast region of signal-to-noise-ratio (SNR), the lower bound of secure multicast rate in (17) is very tight, and has little influence on the energy efficiency of the original problem, which can be seen in Section 4. Therefore, we can reduce the computational complexity while obtaining very approximate solutions of the original problem. The equivalent problem is approximately substituted for the original problem as follows,

(18) $$\begin{array}{cc} \underset{Q}{\arg\max}\;\; & \frac{W{\left[R_{\mathrm{mc}}-R_{\mathrm{ev},\mathrm{ub}}\right]}^{+}}{P}, \end{array}$$

(19) $$\begin{array}{cc} s.t.\; & \mathrm{tr}\left\{Q\right\}\leq P_{\max},\;Q⪰0. \end{array}$$

The feasible solution Q=0 leads the value of $R_{\mathrm{se},\mathrm{lb}}$ to zero. Therefore, all other feasible solutions leading to negative secure multicast rate are not the optimal solutions of the problem in (18). Besides, SNR is defined as $P_{\max}$ in this paper. As a result, we omit the operator ${\left[\cdot \right]}^{+}$ without loss of any optimality, leading the problem equivalent to follows,

(20) $$\begin{array}{cc} \underset{Q}{\arg\max}\;\; & \frac{W(R_{\mathrm{mc}}-R_{\mathrm{ev},\mathrm{ub}})}{P}, \end{array}$$

(21) $$\begin{array}{cc} s.t.\; & \mathrm{tr}\left\{Q\right\}\leq P_{\max},\;Q⪰0. \end{array}$$

We decompose the covariance matrix as $Q=\Phi \Lambda \Phi^{H}$. The columns of Φ and diagonal elements of Λ, respectively, denote the eigenvectors and the eigenvalues of Q, which indicates the multicast signaling directions and the powers allocation. We investigate the eigenvectors of the covariance matrix in Theorem 1.

**Theorem** **1.** *Columns of V are expressed as the eigenvectors of the transmit covariance matrix $Q^{\mathrm{opt}}$ in (20) as follows,*

(22) $$\begin{array}{c} Q^{\mathrm{opt}}=V\Lambda V^{H}. \end{array}$$

**Proof.**  Please refer to the Appendix A. □

According to Theorem 1, we can indicate that the energy efficiency oriented secure multicast transmit signal directions are consistent with the eigenvector of the BS transmission correlation matrix. Therefore, the optimal problem for maximizing the system energy efficiency of secure downlink multicast transmission can be simplified into a beam domain power allocation problem. In particular, the precoding optimization problem of energy efficiency multicast transmission in (20) can be simplified to the following question without loss of optimality,

(23) $$\begin{array}{cc} \underset{\Lambda }{\arg\max}\; & W\frac{\left(R_{\mathrm{mc}}\left(\Lambda \right)-R_{\mathrm{ev},\mathrm{ub}}\left(\Lambda \right)\right)}{P(\Lambda )},\\ s.t.\; & \mathrm{tr}\left\{\Lambda \right\}\leq P_{\max},\;\Lambda ⪰0,\;\Lambda \;\mathrm{diagonal}, \end{array}$$

where

(24) $$\begin{array}{cc} R_{\mathrm{mc}}\left(\Lambda \right)\; & ≜\min_{k}\;R_{k}\left(\Lambda \right), \end{array}$$

(25) $$\begin{array}{cc} R_{k}\left(\Lambda \right)\; & ≜E\left\{\log\det\left\{I_{N_{r}}+G_{k}\Lambda G_{k}^{H}\right\}\right\}, \end{array}$$

(26) $$\begin{array}{cc} R_{\mathrm{ev},\mathrm{ub}}\left(\Lambda \right) & ≜\log\det\left\{I_{N_{e}}+A_{\mathrm{ev}}\left(\Lambda \right)\right\}, \end{array}$$

(27) $$\begin{array}{cc} P\left(\Lambda \right) & ≜\zeta \mathrm{tr}\left\{\Lambda \right\}+MP_{c}+P_{s}. \end{array}$$

In (24), we adopt the minimum transmission rate received by legitimate users into our model to meet the requirement of confidentiality.

### 3.1. Minorize–Maximize Method

We note that function $R_{\mathrm{mc}}\left(\Lambda \right)-R_{\mathrm{ev},\mathrm{ub}}\left(\Lambda \right)$ in (23) is the subtraction of two concave functions. The function is defined as the difference of convex functions (d.c.), which can not be solved by convex optimization directly. Via using the MM method, we adopt an iterative algorithm to deal with it [40,41]. The basic idea is to approximate $R_{\mathrm{ev},\mathrm{ub}}\left(\Lambda \right)$ by the first-order Taylor series expansion and transform it into a linear function. We can solve the subproblem with further algorithm and get the next iteration. The iterative subproblem is as follows,

(28) $$\begin{array}{cc} \Lambda^{\left(\ell +1\right)}=\underset{\Lambda }{\arg\max}\; & W\frac{R_{\mathrm{mc}}\left(\Lambda \right)-\mathrm{tr}\left\{{\left(\frac{\partial }{\partial \Lambda }R_{\mathrm{ev},\mathrm{ub}}\left(\Lambda^{\left(\ell \right)}\right)\right)}^{T}\Lambda \right\}}{P(\Lambda )},\\ s.t.\; & \mathrm{tr}\left\{\Lambda \right\}\leq P_{\max},\;\Lambda ⪰0,\;\Lambda \;\mathrm{diagonal}, \end{array}$$

where *ℓ* denotes the iteration index. Note that the gradient of $R_{\mathrm{ev},\mathrm{ub}}\left(\Lambda \right)$ over Λ can be proved to be a diagonal matrix. We can get its *k*th diagonal element as follows,   

(29) $$\begin{array}{c} {\left[\frac{\partial }{\partial \Lambda }R_{\mathrm{ev},\mathrm{ub}}\left(\Lambda^{\left(\ell \right)}\right)\right]}_{k,k}=\sum_{i=1}^{N_{e}}\frac{{\left[\Omega_{\mathrm{ev}}\right]}_{i,k}}{1+\sum_{j=1}^{M}{\left[\Omega_{\mathrm{ev}}\right]}_{i,j}{\left[\Lambda^{\left(\ell \right)}\right]}_{j,j}}, \end{array}$$

where ${\left\{\Lambda^{\left(\ell \right)}\right\}}_{\ell =0}^{\infty }$ is by the MM method in (28), which can converge to a locally optimal solution of the original problem in (20) [42]. Therefore, our next step is to solve the subproblem of each iteration.

### 3.2. Dinkelbach’s Transform

At the *ℓ* th iteration of subproblem in (28), the fractional function of the objective is still difficult to solve with convex optimition directly. We have $R_{\mathrm{ee}}\left(\Lambda \right)=R_{\mathrm{mc}}\left(\Lambda \right)-\mathrm{tr}\left\{{\left(\frac{\partial }{\partial \Lambda }R_{\mathrm{ev},\mathrm{ub}}\left(\Lambda^{\left(\ell \right)}\right)\right)}^{T}\Lambda \right\}$, which is a concave-minus-linear function. Thus, $R_{\mathrm{ee}}\left(\Lambda \right)$ is a concave functions with respect to Λ. Meanwhile, P(Λ) is a linear function. Dinkelbach’s transform is usually used to approach such concave-linear fractional program, whose advantage is that it does not have to include additional constraints. It has been proved that this algorithm possessed superlinear convergence [43,44]. Besides, Dinkelbach’s transform We express the powers allocation Λ solved in (28) as Γ. Via invoking Dinkelbach’s transform, we can solve the subproblem of each energy efficiency secure multicast optimization in (28) by iteratively solving the sequence of convex optimization problem as follows,

(30) $$\begin{array}{cc} \Gamma^{\left(t+1\right)}=\underset{\Gamma }{\arg\max}\; & WR_{\mathrm{mc}}\left(\Gamma \right)-W\mathrm{tr}\left\{{\left(\frac{\partial }{\partial \Lambda }R_{\mathrm{ev},\mathrm{ub}}\left(\Lambda^{\left(\ell \right)}\right)\right)}^{T}\Gamma \right\}-\eta^{\left(t\right)}P\left(\Gamma \right),\\ s.t.\; & \mathrm{tr}\left\{\Gamma \right\}\leq P_{\max},\;\Gamma ⪰0,\;\Gamma \;\mathrm{diagonal}, \end{array}$$

where *t* denotes the iteration index. $\Lambda^{\left(\ell \right)}$ is the solution of the *ℓ* th outer iteration of the MM method. Note that it is treated as a constant and will not update in the inner iteration in (30). $\eta^{\left(t\right)}$ denotes an auxiliary variable introduced by Dinkelbach’s transform, which can be updated as follows,

(31) $$\begin{array}{c} \eta^{\left(t\right)}=W\frac{R_{\mathrm{mc}}\left(\Gamma^{\left(t\right)}\right)-\mathrm{tr}\left\{{\left(\frac{\partial }{\partial \Lambda }R_{\mathrm{ev},\mathrm{ub}}\left(\Lambda^{\left(\ell \right)}\right)\right)}^{T}\Gamma^{\left(t\right)}\right\}}{P(\Gamma^{\left(t\right)})}. \end{array}$$

By optimizing Γ and η alternately, the generated solution sequence ${\left\{\Gamma^{\left(t\right)}\right\}}_{t=0}^{\infty }$ in (30) is also proved to converge and get the optimal solution of the subproblem in (28).

### 3.3. Deterministic Equivalent

Due to a large number of samples need to be averaged during the expectation operation in (30), the optimization complexity is still high, although each subproblem in (28) is a concave program. Thus, without using Monte Carlo averaging channel realizations, we calculate the deterministic equivalent of the secure transmit rate by adopting the large-dimensional random matrix theory, which can reduce the implementation complexity [45,46]. Note that the method of deterministic equivalent can keep the convexity of the transmission rate [47]. Therefore, the concave subproblem in (28) is given as

(32) $$\begin{array}{cc} \Gamma^{\left(t+1\right)}=\underset{\Gamma }{\arg\max}\; & \min_{k}\;W{\bar{R}}_{k}\left(\Gamma \right)-W\mathrm{tr}\left\{{\left(\frac{\partial }{\partial \Lambda }R_{\mathrm{ev},\mathrm{ub}}\left(\Lambda^{\left(\ell \right)}\right)\right)}^{T}\Gamma \right\}-\eta^{\left(t\right)}P\left(\Gamma \right),\\ s.t.\; & \mathrm{tr}\left\{\Gamma \right\}\leq P_{\max},\;\Gamma ⪰0,\;\Gamma \;\mathrm{diagonal}. \end{array}$$

In (32), ${\bar{R}}_{k}\left(\Gamma \right)$ denotes the deterministic equivalent expression of $R_{k}\left(\Gamma \right)$, which is expressed as

(33) $$\begin{array}{cc} {\bar{R}}_{k}\left(\Gamma \right) & =\log\det\left\{I_{M}+\Xi_{k}\Gamma \right\}+\log\det\left\{{\tilde{\Phi }}_{k}\right\}-\mathrm{tr}\left\{{\tilde{\Xi }}_{k}{\tilde{\Phi }}_{k}^{-1}\right\}, \end{array}$$

where $\Xi_{k}\in C^{M\times M}$, ${\tilde{\Xi }}_{k}\in C^{N_{r}\times N_{r}}$, and ${\tilde{\Phi }}_{k}\in C^{N_{r}\times N_{r}}$ can be obtained by solving the following three fixed-point equations,

(34a) $$\begin{array}{cc} \Xi_{k} & =B_{k}\left({\tilde{\Phi }}_{k}^{-1}\right), \end{array}$$

(34b) $$\begin{array}{cc} {\tilde{\Xi }}_{k} & =C_{k}\left(\Gamma {\left(I_{M}+\Gamma \Xi_{k}\right)}^{-1}\right), \end{array}$$

(34c) $$\begin{array}{cc} {\tilde{\Phi }}_{k} & =I_{N_{r}}+{\tilde{\Xi }}_{k}, \end{array}$$

where $B_{k}\left(X\right)≜E\left\{G_{k}^{H}XG_{k}\right\}\in C^{M\times M}$ and $C_{k}\left(X\right)≜E\left\{G_{k}XG_{k}^{H}\right\}\in C^{N_{r}\times N_{r}}$ are matrix-valued functions, which can output matrices in diagonal forms, the corresponding *i*th diagonal elements are respectively obtained by

(35) $$\begin{array}{cc} {\left[B_{k}\left(X\right)\right]}_{i,i} & =\mathrm{tr}\left\{\mathrm{diag}\left\{{\left[\Omega_{k}\right]}_{:,i}\right\}X\right\}, \end{array}$$

(36) $$\begin{array}{cc} {\left[C_{k}\left(X\right)\right]}_{i,i} & =\mathrm{tr}\left\{\mathrm{diag}\left\{{\left({\left[\Omega_{k}\right]}_{i,:}\right)}^{T}\right\}X\right\}. \end{array}$$

Via the deterministic equivalent approach instead of exhaustive averaging, the expression ${\bar{R}}_{k}\left(\Gamma \right)$ is efficiently calculated by adopting the channel statistics $\Omega_{k}$ in a small number of fixed-point iterations. As a result, the computational complexity to optimize the convex program in (28) can be reduced significantly.

### 3.4. Discussion

In the case of small numbers of antennas, the deterministic equivalent expression ${\bar{R}}_{k}\left(\Gamma \right)$ and $R_{k}\left(\Gamma \right)$ are approximately equal [45,46]. Besides, with respect to Γ, ${\bar{R}}_{k}\left(\Gamma \right)$ is still observed to be concave from (33). Thus, each subproblem in (32) does not change the convexity and still guarantees the convergence. Finally, we obtain our algorithm by two-level iteration. The optimal power allocation is obtained by the inner iteration and assigned to the outer iteration. Then, the initial power allocation of the inner layer is updated after the outer iteration. The proposed energy efficiency optimization algorithm of massive MIMO secure multicast transmission is described in detail in Algorithm 1.
**Algorithm 1** Energy Efficiency Optimization Algorithm for Secure Multicast Transmission**Input:** The beam domain channel transmit statistics CSI $\Omega_{k}$ and $\Omega_{\mathrm{ev}}$, initial power allocation $\Lambda^{\left(0\right)}$, the threshold ϵ**Output:** Optimal power allocation matrix Λ1: Initialization: ℓ=02: Calculate $\Lambda^{\left(0\right)}$ using (26), (27), and (33)3: **repeat**4:    Update ℓ←ℓ+15:    Initialization: t=0, $\Gamma^{\left(0\right)}=\Lambda^{\left(\ell -1\right)}$, $\eta^{\left(0\right)}$ using (31)6:    **repeat**7:     Update t←t+18:     Calculate $\Gamma^{\left(t\right)}$ by using (30) with $\eta^{\left(t-1\right)}$9:     Update $\eta^{\left(t\right)}$ using (31)10:    **until**
$∥\Gamma^{\left(t\right)}-\Gamma^{\left(t-1\right)}∥\leq \epsilon$11:    Return $\Lambda^{\left(\ell \right)}=\Gamma^{\left(t\right)}$12: **until**
$∥\Lambda^{\left(\ell \right)}-\Lambda^{\left(\ell -1\right)}∥\leq \epsilon$13: Return $\Lambda =\Lambda^{\left(\ell \right)}$

## 4. Simulation Results

To illustrate the performance of the energy efficiency optimization for massive MIMO secure multicast transmission with known statistical CSI. A series of assumptions about the simulation environment, transmission channel and antenna configuration are made in our work [48]. The major parameters related to simulation, such as the power consumption, are listed in Table 1.

**Figure 1:**
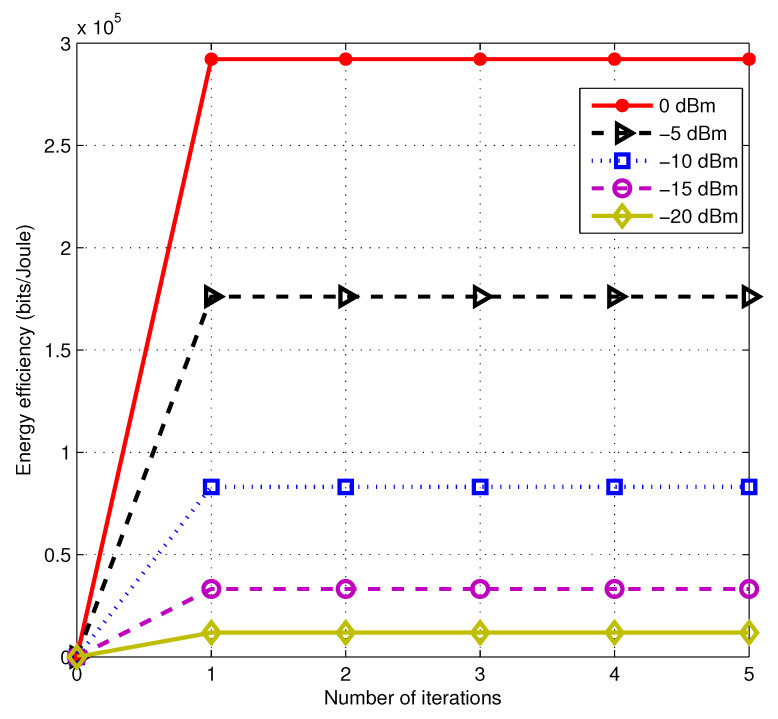
The convergence performance of outer iteration of Algorithm 1 under different signal-to-noise-ratios (SNRs).

First of all, we evaluate our proposed algorithm convergence performance. In order to observe the convergence of the whole algorithm, we choose the outer iteration to draw Figure 1, which plots energy efficiency versus the number of iterations under different SNR. From Figure 1, it can be observed that our algorithm exhibits fast convergence performance. Besides, the lower the SNR regime is, the quicker the iteration converges. Thus, we can indicate that power allocation might perform better under the high SNR regime on optimizing secure multicast energy efficiency. In the regime of Figure 1, with the SNR increasing, the gain of energy efficiency increases faster, which shows that the secure multicast rate is growing rapidly.

**Figure 2:**
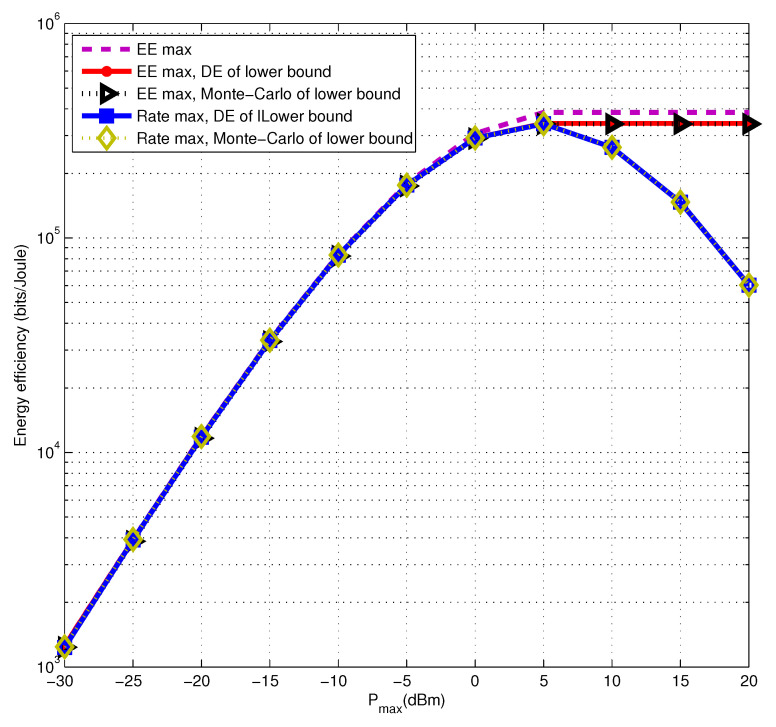
The lower bound of energy efficiency by energy efficiency maximization and rate maximizations algorithm via using the deterministic equivalent and Monte Carlo methods, compared with the original energy efficiency.

Next, we evaluate the energy efficiency performance of the proposed algorithm with a lower bound of secure multicast rate and the original optimization problem with the secure rate obtained by the Monte Carlo Method. It is shown in Figure 2 that the solutions of the two are quite tight. Therefore, we can handle the approximation problem to obtain the desired energy efficiency but significantly reduce the algorithm complexity. Then, we compare the energy efficiency performance optimized by the proposed algorithm with the traditional rate maximization one. In addition, the deterministic equivalent results are compared with those of the Monte Carlo method. According to Figure 2, it is not difficult to find that the gap between the deterministic equivalent and the Monte Carlo methods is almost zero. Besides, under the low transmit power regime, our proposed energy efficiency maximization and the traditional rate maximization algorithms have similar energy efficiency performance. It is also proved that the inference in Figure 1 is correct. However, under the high transmit power regime, the energy efficiency maximization algorithm is obviously superior to the traditional rate maximization one. It also shows that we can find a compromise power allocation to meet the two requirements at the same time in the goal of maximum rate and maximum energy efficiency for massive MIMO secure multicast transmission.

**Figure 3:**
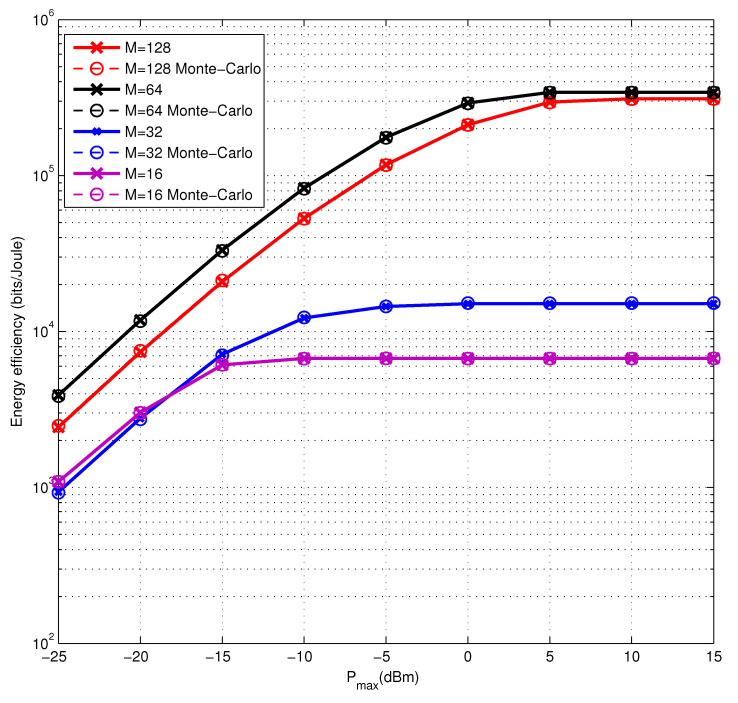
The energy efficiency performance of the proposed algorithm and Monte Carlo method with the numbers of BS antennas varying from M=16 to M=128.

Finally, we study the energy efficiency performance of our algorithm versus the numbers of antennas at the BS and show the results in Figure 3. With the number of BS antennas increasing, the change of energy efficiency performance tends to be flat, which shows that the power consumption dominates the performance of energy efficiency when the number of BS antennas is large. Besides, it can be indicated that we can find a suitable power allocation to meet our requirement of high energy efficiency. At the same time, we can also find that the energy efficiency does not increase with the number of antennas. This is because under large antennas and low power constraint, the secure rate changes faster than power consumption at the BS with the increase of the maximum power constraint, while the dynamic power consumption of the BS antennas increases rapidly under the high power constraint, leading the situation on the contrary. Finally, when the number of antennas increases, the system needs more power to reach better energy efficiency. Besides, the energy efficiency performance of the Monte Carlo method is quite close to the proposed one, which confirms the accuracy of the analysis.

## 5. Conclusions

We studied the energy efficiency optimization problem in downlink secure multicast transmission for massive MIMO with known statistical CSI in this paper. The energy efficiency maximization problem was formulated as maximizing the ratio of the secure multicast rate to the power consumption. We then obtained the secure multicast rate by introducing its lower bound to simplify the objective problem. Via making the transmit signaling directions into a closed-form, we simplified the energy efficiency optimization of matrix-valued strategy design to a beam domain power allocation problem. Next, we designed an algorithm including two-level iteration in the beam domain, and guaranteed the algorithm to converge to a locally optimal point based on the MM method and Dinkelbach’s transform. In addition, via substituting the multicast rate for its deterministic equivalent, the complexity of computational was significantly reduced. We made extensive simulations to verify the effectiveness of our algorithm. A large number of simulation results showed that the algorithm has fast convergence and low complexity. Compared with the rate maximization method, the proposed algorithm had more efficient and stable performance under the high transmission power constraint.

## Figures and Tables

**Table 1:** Major simulation parameters of massive multiple-input multiple-output (MIMO) secure multicast transmission.

Parameter	Value
Channel model	3GPP SCM
Array topology	ULA with half wavelength antenna spacing
Propagation scenario	Suburban macro-cell
The numbers of BS antennas	*M* = 64
The numbers of UTs	*K* = 8
The numbers of UTs antennas	$N_{r}$ = 4 (∀k)
The numbers of eavesdropper antennas	$N_{e}$ = 4
Bandwidth	*W* = 10 MHz
Amplifier inefficiency factor	ξ = 5
Static power consumption	$P_{s}$ = 40 dBm
Hardware dissipated power per antenna	$P_{c}$ = 30 dBm

## Appendix A. Proof of Theorem 1

We can observe that the upper bound of the eavesdropper transmit rate $R_{\mathrm{ev},\mathrm{ub}}$ in (15) is irrelevant to the off-diagonal elements of $V^{H}QV$. Then, we define $\tilde{Q}=V^{H}QV$. The multicast rate of UTs $R_{\mathrm{se}}$ in (20) can be expressed as follows,

(A1) $$\begin{array}{c} R_{\mathrm{se}}=\min_{k}\;E\left\{\log\det\left\{I_{N_{r}}+G_{k}\tilde{Q}G_{k}^{H}\right\}\right\}-R_{\mathrm{ev},\mathrm{ub}}\left(\tilde{Q}\right), \end{array}$$

Following a similar line of reasoning of the proof in [49], define $D_{r}\in R^{N_{r}\times N_{r}}$ as a diagonal matrix whose diagonal entries are all 1 except the (r,r)th entry being −1. Please note that the matrix $D_{r}$ is an unitary matrix, which means $G_{k}D_{r}$ has the same distribution as $G_{k}$ [49], thus

(A2) $$\begin{array}{cc} \; & R_{\mathrm{se}}\left(D_{r}\tilde{Q}D_{r}^{H}\right)\\ = & \min_{k}\;E\left\{\log\det\left\{I_{N_{r}}+G_{k}D_{r}\tilde{Q}D_{r}^{H}G_{k}^{H}\right\}\right\}-R_{\mathrm{ev},\mathrm{ub}}\left(D_{r}\tilde{Q}D_{r}^{H}\right)\\ = & \min_{k}\;E\left\{\log\det\left\{I_{N_{r}}+G_{k}\tilde{Q}G_{k}^{H}\right\}\right\}-R_{\mathrm{ev},\mathrm{ub}}\left(\tilde{Q}\right)\\ = & R_{\mathrm{se}}\left(\tilde{Q}\right). \end{array}$$

Here, $\tilde{Q}$ is equal to $D_{r}\tilde{Q}D_{r}^{H}$ expect for the off-diagonal entries in the *r*th row and the *r*th column, whose signs are reverse. We want to prove that nulling all the off-diagonal entries of $\tilde{Q}$ in the *r*-th row and the *r*-th column will not decrease the transmit rate with respect to $\tilde{Q}$. We invoke Jensen’s inequality to prove it.

(A3) $$\begin{array}{cc} \; & R_{\mathrm{se}}\left(\frac{1}{2}\left(\tilde{Q}+D_{r}\tilde{Q}D_{r}^{H}\right)\right)\\ = & \min_{k}\;E\left\{\log\det\left\{I_{N_{r}}+\frac{1}{2}\left(G_{k}\left(\tilde{Q}+D_{r}\tilde{Q}D_{r}^{H}\right)G_{k}^{H}\right)\right\}\right\}-R_{\mathrm{ev},\mathrm{ub}}\left(\frac{1}{2}\left(\tilde{Q}+D_{r}\tilde{Q}D_{r}^{H}\right)\right)\\ \geq & \min_{k}\left(\frac{1}{2}E\left\{\log\det\left\{I_{N_{r}}+G_{k}\tilde{Q}G_{k}^{H}\right\}\right\}\right)-\frac{1}{2}R_{\mathrm{ev},\mathrm{ub}}\left(\tilde{Q}\right)\\ + & \min_{k}\left(\frac{1}{2}E\left\{\log\det\left\{I_{N_{r}}+G_{k}D_{r}\tilde{Q}D_{r}^{H}G_{k}^{H}\right\}\right\}\right)-\frac{1}{2}R_{\mathrm{ev},\mathrm{ub}}\left(D_{r}\tilde{Q}D_{r}^{H}\right)\\ = & \frac{1}{2}R_{\mathrm{se}}\left(\tilde{Q}\right)+\frac{1}{2}R_{\mathrm{se}}\left(D_{r}\tilde{Q}D_{r}^{H}\right)\\ = & R_{\mathrm{se}}\left(\tilde{Q}\right). \end{array}$$

We repeat the *r* for 1 to $N_{r}$, and find that the numerator of the problem is maximized when $\tilde{Q}$ is diagonal. Meanwhile, the consumed power $P≜\zeta \mathrm{tr}\left\{Q\right\}+MP_{c}+P_{s}$ is irrelevant to the off-diagonal elements of $V^{H}QV$. Thus, the objective problem in (20) is maximized when $\tilde{Q}=V^{H}QV=V^{H}\Phi \Lambda \Phi^{H}V$ is diagonal. This concludes the proof.
